# Effect of multi-system polishing on shear bond strength of nanohybrid composites to enamel: an *in vitro* study

**DOI:** 10.7717/peerj.21549

**Published:** 2026-08-24

**Authors:** Ali Alsaeed, Suraj Arora, Basil M. Rubayan, Khalid M. Abdelaziz, Mohammed A. Alshahrani, Ayman M. Fae, Musleh AlShahrani, Abdulrahman Yahya Alshahrani, Anuj Bhardwaj, Swadheena Patro, Ajinkya M. Pawar

**Affiliations:** 1 Department of Restorative Dental Sciences, College of Dentistry, King Khalid University, Abha, Saudi Arabia; 2 Department of Oral and Dental Surgery, College of Dentistry, King Khalid University, Abha, Saudi Arabia; 3 Shubbah Primary Health Care, Aseer Health Cluster, Ministry of Health, Khamis Mushait, Saudi Arabia; 4 Department of Conservative Dentistry and Endodontics, College of Dental Science and Hospital, Indore, Madhya Pradesh, India; 5 Department of Conservative Dentistry and Endodontics, Kalinga Institute of Dental Sciences, KIIT University, Bhubaneswar, Odisha, India; 6 Conservative Dentistry and Endodontics, Nair Hospital Dental College, Maharashtra University of Health Sciences, Mumbai, Maharashtra, India

**Keywords:** Dental polishing, Shear strength, Dental enamel, Composite resins, Dental bonding, Dental adhesives

## Abstract

**Background:**

The purpose of this study was to compare the influence of three enamel finishing and polishing systems (Sof-Lex discs, lithium disilicate Eve burs and lithium disilicate polishing burs) on the shear bond strength and distribution of failure modes of nanohybrid composite resin bonded to enamel.

**Materials and Methods:**

A sample of 240 healthy human premolars were randomized into four treatment groups (*n* = 60 per group): (1) untreated control, (2) Sof-Lex disc polishing, (3) lithium disilicate EVE burs (Li-EVE) burs, and (4) lithium disilicate polishing burs (Li-Polish) burs. Specimens were prepared with standardized protocols and restored with a nanohybrid composite and universal adhesive system. The enamel surfaces were etched with 35% phosphoric acid. Shear bond strength (SBS) was measured using a universal testing machine and fracture modes were assessed with a dissecting microscope. Descriptive statistics, one-way analysis of variance (ANOVA), Games–Howell *post hoc* test, and chi-square analysis of the mode of failure were conducted at α = 0.05.

**Results:**

There were significant differences in SBS between the groups (F(3,236) = 10.54, *p* = 1.6 ± 10^−6^). Both Sof-Lex (31.39 ± 7.81 MPa) and Li-Polish (30.94 ± 5.70 MPa) had significantly higher bond strength values than control (23.18 ± 14.04 MPa) and Li-EVE (26.00 ± 8.26 MPa) (*p* ≤ 0.024). There was no significant difference between Sof-Lex and Li-Polish (p = 0.82). The failure modes were predominantly cohesive in all the groups and the distribution of failure modes was not significantly different (*χ*^2^ (3) = 6.31, *p* = 0.098, Cramér’s V = 0.162).

**Conclusion:**

The enamel polishing system significantly influenced composite bond strength. Multi-step polishing systems, *e.g.*, Sof-Lex discs or Li-Polish burs, increased adhesive performance markedly over the control groups or one-step polished systems to untreated enamel. The failure mode was not significantly influenced by polishing systems. These findings demonstrated the importance of finishing systems for successful restorative performance.

## Introduction

Modern restorative dentistry relies heavily on the use of direct resin composites; these materials provide excellent aesthetics, conserve healthy tooth structure when being applied to a tooth, and provide a high degree of adhesion or bond strength to both enamel and dentin. Though there have been many developments in adhesive systems, the longevity or success of composite restorations is dependent on the health of the tooth/composite interface over time. Failures such as loss of marginal discolouration, microleakage, and/or secondary caries are sometimes due to inadequate bonding, which is often associated with a compromised adhesive bond at the enamel surface (Elgezawi et al., 2022). Thus, optimizing the enamel substrate prior to adhesive bonding is a critical component of clinical success.

Adhesion to enamel is governed not only by adhesive chemistry but also by the condition of the substrate at the time of bonding. The creation of an appropriate surface microtopography through acid etching enables micromechanical interlocking *via* resin tag formation, while surface cleanliness and free energy influence adhesive wetting and infiltration (Perdigão, 2020; Sato et al., 2021). These factors collectively determine the quality and durability of the adhesive interface. Accordingly, procedures performed before bonding—particularly finishing and polishing—may significantly alter enamel surface characteristics and thereby affect bond strength.

Phosphoric acid etching is a well-known method for changing the surface texture of enamel by creating micro pores on the enamel surface that provide mechanical anchorage through micro-mechanical retention mechanisms (Han, Liang & Xie, 2021). The condition of the enamel surface is extremely important prior to etching (Kadhim, Deb & Ibrahim, 2023). Finishing/polishing techniques can change the structure of the surface by removing aprismatic enamel, changing the smear layer and changing both the uniformity and cleanliness of the surface. These surface changes will also affect the depth and pattern of acid etching, and also the degree to which resin will infiltrate and form tags after acid etching (Okumuş et al., 2023). As such, even when etch and rinse protocols are used for adhesive bonding, the pre-etch surface conditions produced by different polishing systems will greatly affect the overall bonding characteristics of a bonded tooth surface.

Routine finishing and polishing are two types of procedures carried out on a regular basis during restorative and orthodontic treatment to achieve smooth and uniform surfaces of enamel after the preparation process and/or removal of adherent materials. Among the various systems available, the multistep aluminum oxide disc sequences (*e.g.*, Sof-Lex) are frequently used as reference systems because they consistently yield very smooth, uniform, and easily damaged surfaces of enamel (Shah et al., 2019; Křivková et al., 2023). Clinical and experimental evidence supports the efficacy of these systems for providing substrate surfaces suitable for creating etching patterns prior to bonding, and for optimizing bonding conditions.

In addition to disc-based systems, various rotary polishing instruments, including diamond-impregnated burs and ceramic polishing kits, are commonly used in clinical practice. Although they are frequently picked because they do their job very well and are simple to use, it is not yet known what effect they might have on enamel surface properties and later on adhesive performance. In contrast to multi-step polishing systems (*i.e.,* disc systems) that have different levels of fineness, as the surfaces become polished and smoother, rotary polishing will create surface textures that are based upon the type of abrasive used, its dimensions, and how the polisher has been applied (Jurado et al., 2021). These variable surface textures may change the microtopography of the enamel and its wettability, therefore, how well the resin will penetrate into the enamel and how well the resin will bond with the enamel. Notably, lithium-disilicate polishing systems are primarily designed for ceramic substrates, which differ from enamel in structure and hardness. As a result, their abrasive composition and polishing dynamics may interact differently with the enamel prism architecture, potentially producing distinct surface morphologies compared to conventional aluminum-oxide disc systems (Jurado et al., 2023).

There is a growing interest in this area; however, the available evidence is still inconsistent. Some studies have reported that the use of multi-step polishing systems produces a better bond to enamel than the use of simplified or rotary polishing techniques; on the other hand, other studies have shown no significant difference between these two techniques when using the same adhesive protocols (Křivková et al., 2023; Lippert et al., 2024; Bulut et al., 2025). The dissimilarities in study findings may be due to variability in the study designs including differences in enamel substrate conditions, polishing parameters (such as pressure, speed, and duration), and bonding strategies (Kanniappan, Hari & Jujare, 2022). Therefore, the effect of specific polishing systems on the bonding of enamel is still not definitively determined.

Clinicians often choose finishing and polishing systems based on either how easy or convenient they are, as well as how familiar they are even if the two surfaces have not previously been used for adhesive bonding. The reason this is important is that there have not been enough direct comparative studies done to demonstrate how different polishing protocols affect the enamel surfaces so they will offer the best chance of providing the optimal bond for attaching your dental crowns. Since these studies have not been conducted, and subtle differences in the enamel surface preparation may be enough to significantly impact the bond strength or longevity of your dental restoration, this represents a significant gap in the literature.

Therefore, the present *in vitro* study was designed to evaluate the effect of commonly used finishing and polishing systems on the shear bond strength of nanohybrid composite resin to enamel. Specifically, enamel surfaces were prepared using either a multistep aluminum-oxide disc system (Sof-Lex), lithium disilicate–based polishing systems (Li-EVE and Li-Polish), or left unpolished as a control. All specimens were bonded using a standardized etch-and-rinse protocol with a universal adhesive to isolate the effect of surface preparation.

The primary objective was to compare shear bond strength across these surface-conditioning approaches. A secondary objective was to assess the distribution of failure modes to provide insight into the nature of the adhesive interface. The null hypotheses were that (1) no significant differences in bond strength would exist among the different polishing systems and (2) polishing protocols would not result in statistically significant differences in failure mode distribution under controlled *in vitro* conditions.

## Materials & Methods

### Study design and ethical approval

This was an *in vitro* randomized comparative study assessing the impact of three finishing/polishing protocols on enamel-composite adhesion. Ethical approval was obtained from the Institutional Ethics Committee of College of Dental Science and Hospital CDSH/Admin/2025/107 dated 11-04-2025. The informed consent was waived by ethics committee because the study was conducted entirely *in vitro* using extracted human teeth that were collected after routine clinical extraction procedures and were not extracted specifically for the purpose of this study. No direct intervention was performed on human participants, no biological samples were linked to identifiable patient information, and no personal, demographic, or clinical data were recorded or analyzed.

### Sample size estimation

A priori calculation of the anticipated sample size was based upon shear bond strength (SBS) as our primary outcome measure. Because there are few studies in the literature that directly compare adhesive bond strength with different surface treatments and adhesive protocols in restorative *in vitro* studies, we used the most recent study evaluating SBS for universal nanohybrid composite resin repair to guide us in estimating the effect sizes (Ömeroğlu et al., 2025). In this study, the mean SBS values of approximately 14–23 MPa between groups using one-way analysis of variance (ANOVA) and two-way ANOVA designs. Researchers were able to estimate the minimum total sample size for those studies using a moderate effect size (*f* = 0.25), a significance level (*rmα*) of 0.05 and 90% desired statistical power for 4 group comparisons (43 specimens per group). As a result of estimating the minimum total sample size to be 172 specimens (43 specimens per group), the study sample size was increased to 240 specimens (60 specimens per group) to allow for potential loss in the sample, variability associated with extracted tooth substrates, and to ultimately improve the precision of the effect estimates. This sample will provide sufficient power to detect clinically meaningful differences in SBS between experimental conditions.

### Tooth selection, eligibility, and storage

Two hundred forty non-carious human premolars, extracted for orthodontic or periodontal reasons, were obtained from donors age 20–35 years. Teeth exhibiting cracks, carious lesions, fluorosis, restorations, or enamel defects were excluded. After debridement, samples were disinfected in 0.5% chloramine at 4–7 °C for 7 days and stored in distilled water at 4–7 °C for up to 3 months prior to testing, in accordance with established guidelines for *in vitro* adhesion studies (Bourgi et al., 2024).

### Randomization and allocation

Teeth were randomly allocated to four groups (*n* = 60 per group) by sealed envelopes: G1, unpolished control; G2, Sof-Lex discs; G3, Li-EVE burs; and G4, Li-polishing burs. Specimens were embedded in gypsum to the cementoenamel junction to secure roots during preparation and testing. Group identifiers were used during data analysis to conceal allocation bias.

### Enamel surface preparation

**Figure 1:**
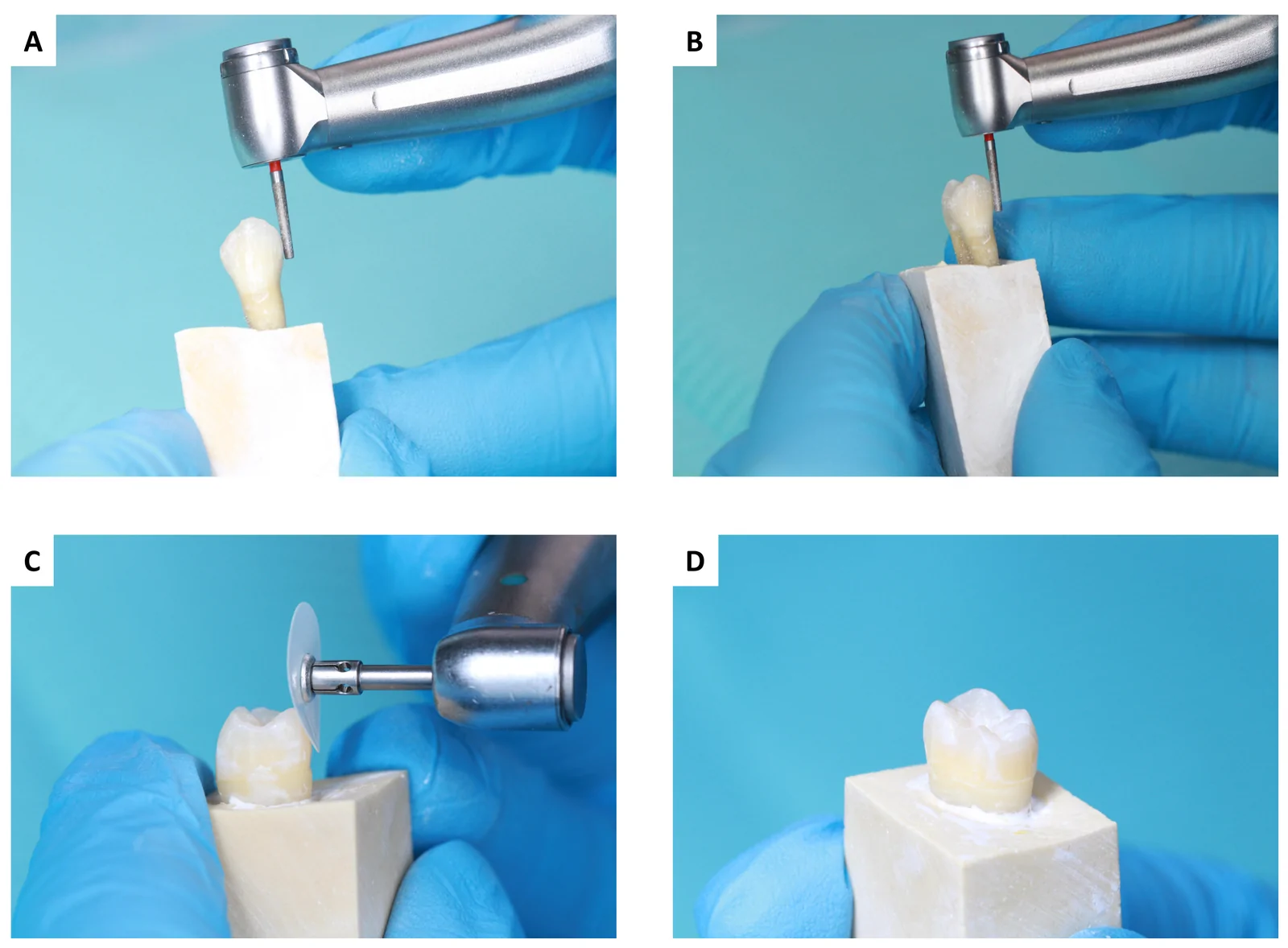
Standardized protocol for tooth surface preparation and finishing. (A) Mesial surface reduction of 0. 3 mm performed using a flat-end diamond bur, (B) Lingual surface reduction of 0.3 mm performed using a flat-end diamond bur, (C) Surface finishing and polishing carried out using the Sof-Lex™ disc system, and (D) Final tooth surface appearance following completion of the polishing procedure.

On each tooth, a flat enamel window was prepared on the mesial surface with a fine flat-ended tapered diamond bur (DENTEX DIAMOND BURS #F306) to create a standardized bonding area without dentin exposure. Each tooth contributed a single standardized enamel surface (mesial surface) and was assigned to only one experimental group. No tooth was used to provide multiple test surfaces across different groups, ensuring statistical independence of specimens (Fig. 1).

All finishing and polishing procedures were performed by a single calibrated operator with prior training in the use of all systems to minimize inter-operator variability. To standardize application pressure, a light, controlled hand pressure was maintained throughout all procedures, consistent with manufacturer recommendations and prior *in vitro* protocols.

**Figure 2:**
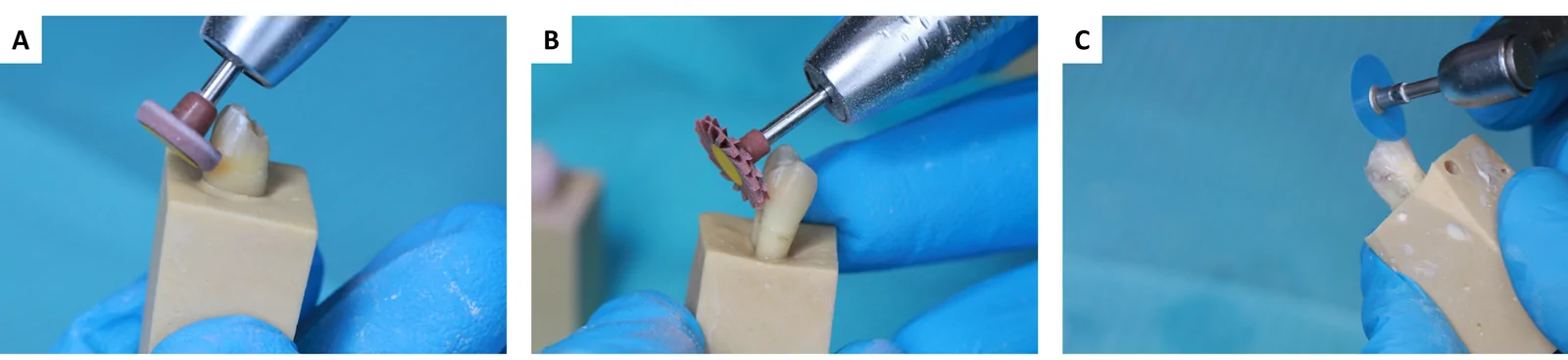
Comparative polishing protocols applied to standardized tooth surfaces. (A) Tooth surface polishing performed using Li-Polish burs, (B) Tooth surface polishing performed using Li-EVE burs, and (C) Tooth surface polishing performed using the Sof-Lex™ disc system.

Finishing and polishing procedures (Fig. 2)

• G1: Control. No finishing or polishing after enamel reduction with a diamond bur.
• G2: Sof-Lex discs. A three-step, aluminum-oxide disc sequence was applied in a water spray—medium, red; fine, yellow; and superfine, white—each step for 20 s at 40,000 RPM, with a 10-seconds water rinse and air dry between each step.
• G3: Li-EVE burs. A fan-shaped, diamond-impregnated polisher was used in two steps, medium, red and fine, gray, each for 20 s at 40,000 revolutions per minute (RPM) in a water spray, with a 10-seconds rinse and air dry between each step.
• G4: Li-polishing burs. The EVE Diasynt/Diapro lithium disilicate polishing kit (HDP8), were used in two steps, coarse, red and fine, green, each for 20 s at the maximum, 40,000 RPM, in a water spray, with a 10-seconds rinse and air dry between each step.

A standardized enamel reduction was performed to create a uniform and flat bonding substrate across all specimens, thereby minimizing variability related to natural enamel curvature, aprismatic enamel layers, and surface irregularities. This approach is commonly adopted in *in vitro* adhesion studies to improve internal validity and allow controlled comparison between experimental groups.

### Adhesive and composite application

**Figure 3:**
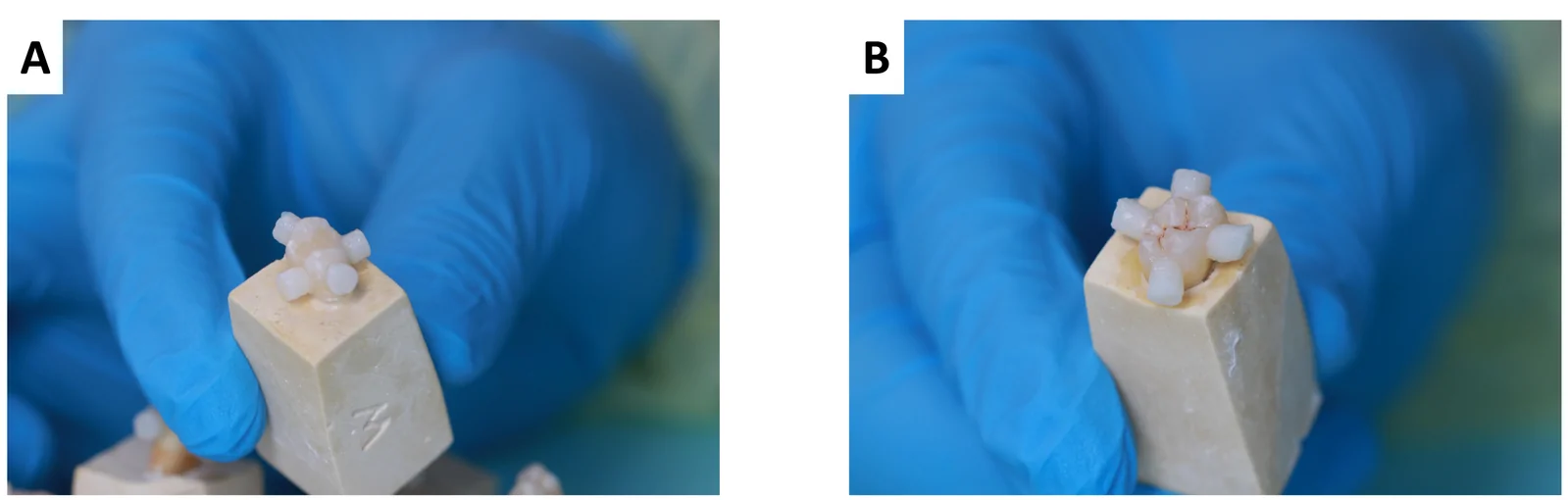
Representative appearance of tooth surfaces following composite restoration. (A) Tooth surface after application of the composite restoration and (B) Tooth surface after application of the composite restoration (alternate view).

All specimens were treated with 35% phosphoric acid for etching for 20 s; after being rinsed for 30 s, they were gently air-dried. A universal adhesive (3M™ Scotchbond™ Universal/Universal Plus) was applied in 2 coats and light-cured with an LED curing unit (3M Elipar™ DeepCure-L) for 10 s. A cylinder-shaped mold (three mm diameter ×3 mm height) was centered on the prepared enamel surface, with visual alignment perpendicular to the surface. The consistent placement of the molds also had a standardized enamel window. All procedures were conducted by one operator trained to reduce variability. Thereafter, a nanohybrid resin composite (Luna by SDI) was placed into each mold cavity and light-cured for 40 s, following the manufacturer’s instructions (Fig. 3).

### Shear bond strength (SBS) testing

**Figure 4:**
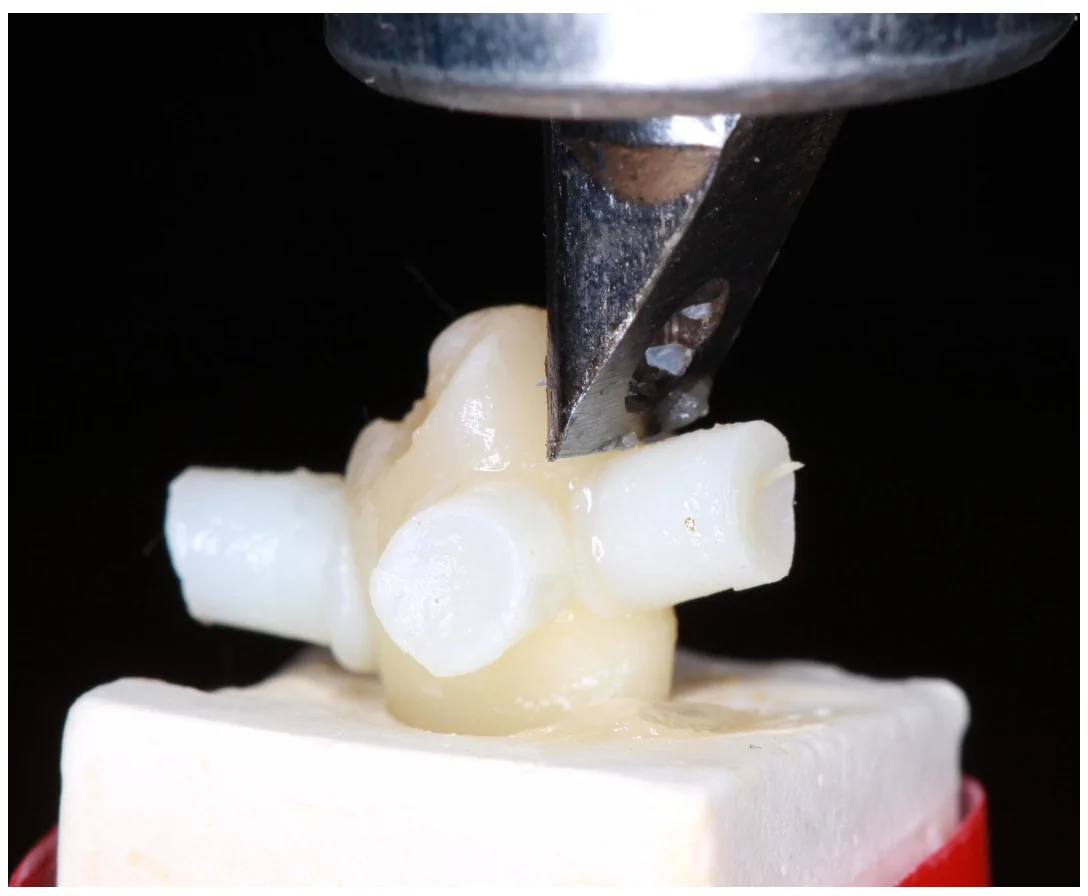
Shear bond strength (SBS) testing of composite restorations. Experimental setup showing the composite restoration mounted in a universal testing machine, with a chisel-shaped loading head positioned parallel to the tooth–composite interface prior to load application.

The specimens were placed into distilled water at 37 °C for 24 h before they were tested. Bond strength was determined using a universal material testing machine. A chisel-shaped loading blade was applied using a crosshead speed of one mm/min at the enamel-composite interface until failure occurred. Chisel loading was chosen because it is commonly used and standardized for comparing adhesive performance between groups of materials using similar *in vitro* test conditions. While macro-shear bond strength testing has a number of limitations (*i.e.,* non-uniform stress distribution, possible cohesive or mixed mode failures), differences among the groups can be made with some level of confidence because standard specimen shapes, areas of bond and loading conditions were used (Fig. 4). The maximum load at which each specimen failed (in N) was measured, and by using this value and the cross-sectional area of the composite cylinder the shear bond strength was calculated (in MPa).

### Failure mode analysis

**Figure 5:**
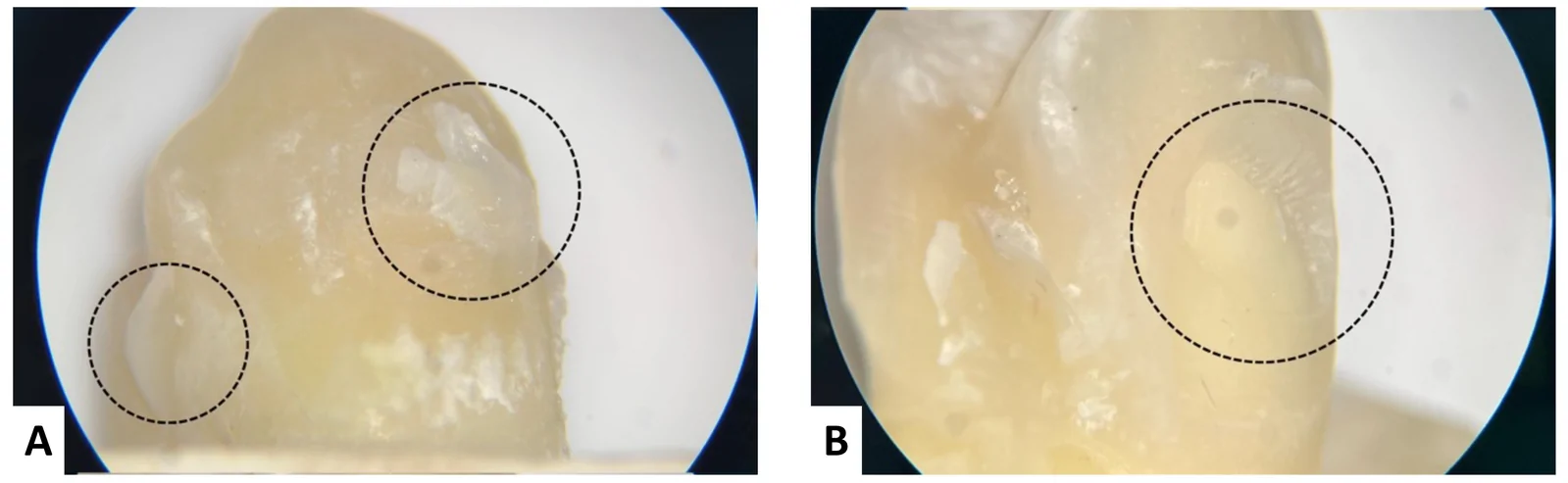
Representative failure modes observed following shear bond strength (SBS) testing. (A) Cohesive failure within the composite resin, characterized by residual composite material remaining adhered to the tooth surface after load application and (B) Cohesive failure within the tooth structure, showing partial dislodgement of tooth substrate along with the bonded composite following load application.

Failure modes were classified as adhesive, cohesive, or mixed using stereomicroscopic evaluation (Fig. 5). All assessments were performed by a single calibrated examiner, and a subset of specimens was re-evaluated to ensure consistency of scoring.

### Statistical analysis

SBS data were expressed as mean ± standard deviation and 95% confidence intervals. Distributional assumptions were verified (normality and homogeneity). Differences between groups were examined *via* one-way ANOVA. Due to heteroscedasticity present in variance diagnostics, pairwise comparisons were conducted using the Games–Howell procedure. An effect size (ω^2^) was also reported for the omnibus test. Failure-mode distributions were assessed with *χ*^2^ and Cramér’s V. To further explore the group effects on failure type, a logistic regression model was developed with failure type (adhesive = 1; cohesive = 0) as the outcome variable and groups as the predictor, using the control group as the referent. The significance level was set at α = 0.05. All analyses were conducted using SPSS v26.

## Results

### Shear bond strength

Table 1 provides descriptive statistics for shear bond strength (SBS). There was a significant difference in bond strength between groups (F(3,236) = 10.54, *p* = 1.6 × 10^−6^, ω^2^ = 0.107; moderate effect size). Control specimens produced the lowest mean SBS (23.18 ± 14.04 MPa), whereas Sof-Lex (31.39 ± 7.81 MPa) and Li-Polish (30.94 ± 5.70 MPa) demonstrated the highest bond strength values. Li-EVE averaged (26.00 ± 8.26 MPa) between Sof-Lex and Li-Polish bond strength values.

**Table 1:** Shear bond strength (SBS) by group with descriptive statistics.

**Group**	**n**	**Mean (MPa)**	**SD (MPa)**	**Median (MPa)**	**95% CI Lower**	**95% CI Upper**
Control	60	23.18	14.04	20.65	19.54	26.82
Li-EVE	60	26.00	8.26	25.71	23.86	28.14
Li-Polish	60	30.94	5.70	31.05	29.48	32.40
Sof-Lex	60	31.39	7.81	31.23	29.38	33.41

**Notes.**

Values are mean ± standard deviation; 95% confidence intervals computed from *t* distribution.

**Figure 6:**
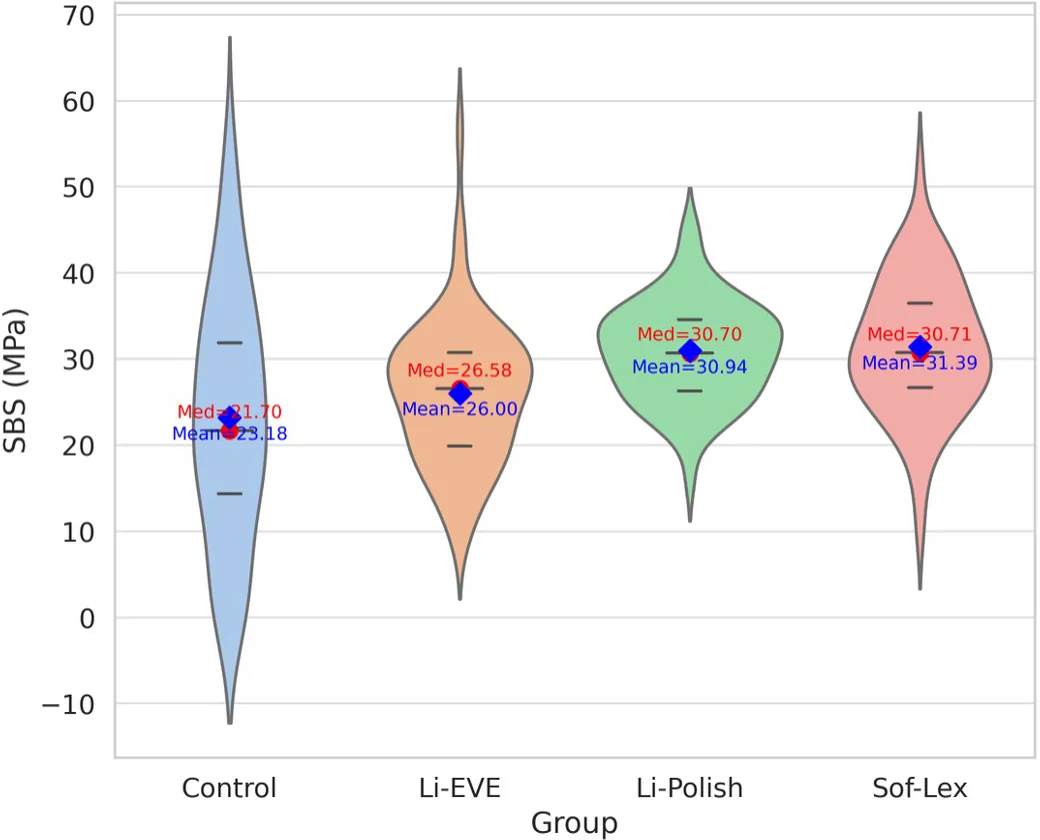
Violin plots of shear bond strength (SBS) for each group (*n* = 60 per group). Median values are indicated by red circles and mean values by blue diamonds, with corresponding numeric labels. Sof-Lex and Li-Polish demonstrated the highest SBS, while control showed the lowest values.

The violin plots in Fig. 6 represent the distribution of SBS data along with the mean and median values. Pairwise Games–Howell comparisons (Table 2) showed that Sof-Lex and Li-Polish had significantly higher SBS than control and Li-EVE (*p* ≤ 0.024), while Sof-Lex and Li-Polish did not differ statistically (*p* = 0.82). Control and Li-EVE also did not differ (*p* = 0.23).

**Figure 7:**
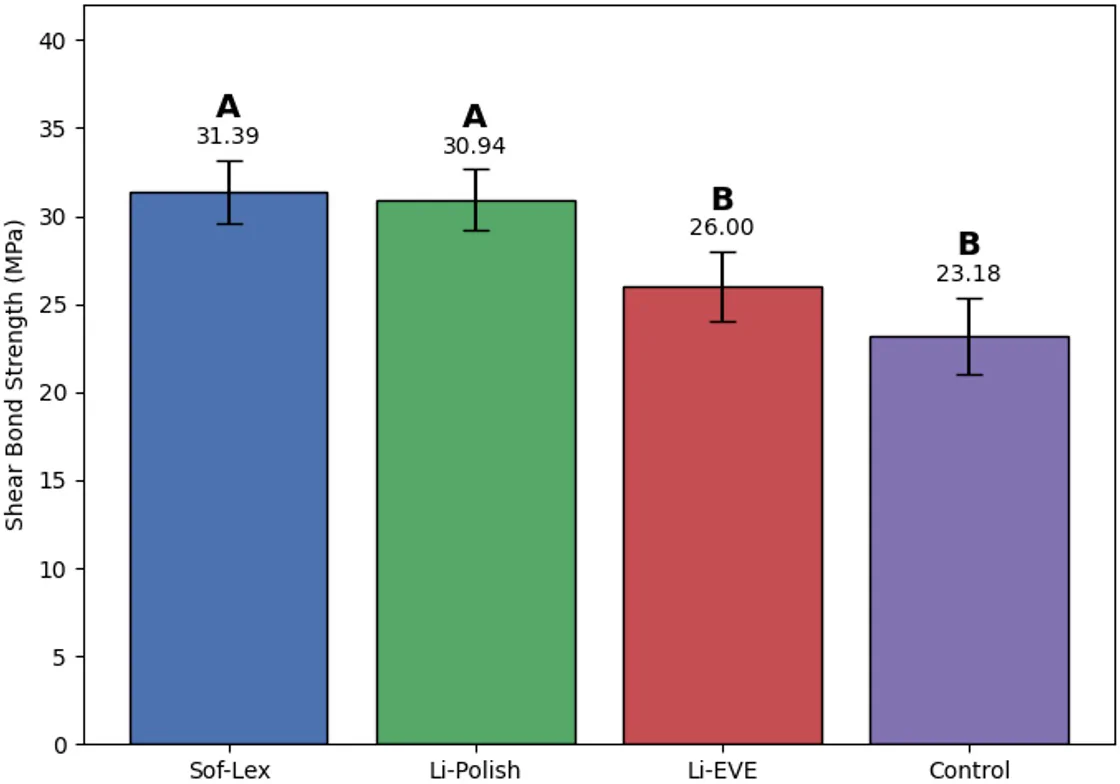
Bar graph showing mean shear bond strength (SBS) values (MPa) with 95% confidence intervals for the different polishing groups. Numerical values indicate group means. Groups sharing the same letter are not significantly different according to the Games–Howell *post hoc* test (α = 0.05). Sof-Lex and Li-Polish (A) demonstrated significantly higher SBS compared with Li-EVE and control (B).

The compact letter display in Fig. 7 shows that Sof-Lex and Li-Polish form one statistically homogeneous group, while control and Li-EVE form another.

### Failure mode distribution

**Figure 8:**
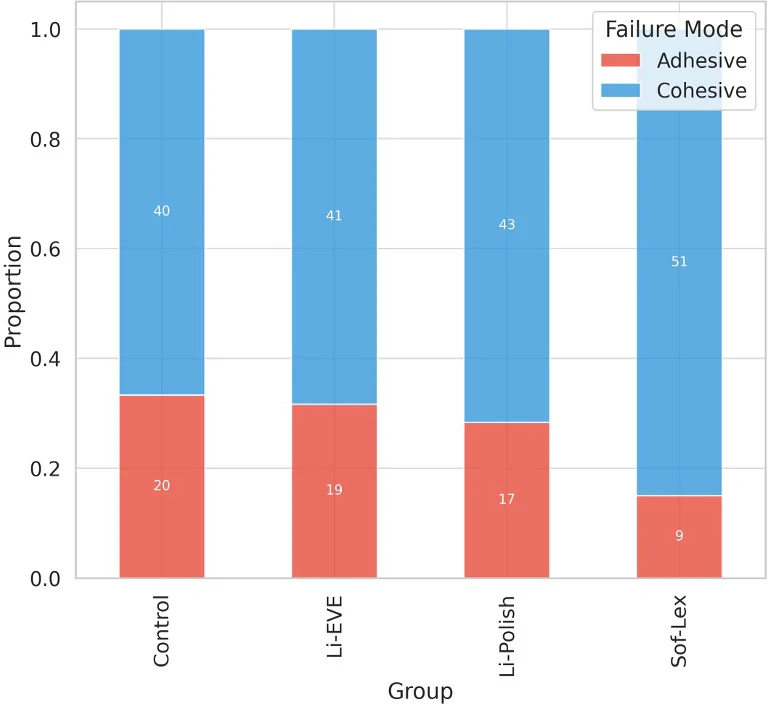
Stacked bar chart of failure mode distribution (adhesive *vs.* cohesive) across polishing groups. Cohesive failures were predominant in all groups.

Failure mode frequencies are indicated in Table 3 and shown in Fig. 8. Cohesive failures were predominant in all groups (from 66.7% for control to 85.0% for Sof-Lex specimens). Chi-squared analysis revealed no significant differences for failure distribution (*χ*^2^(3) = 6.31, *p* = 0.098, Cramér’s V = 0.162), and logistic regression also confirmed there were no significant group effects. Therefore, while differences of polishing system impacted bond strength, it did not change the type of failure.

**Figure 9:**
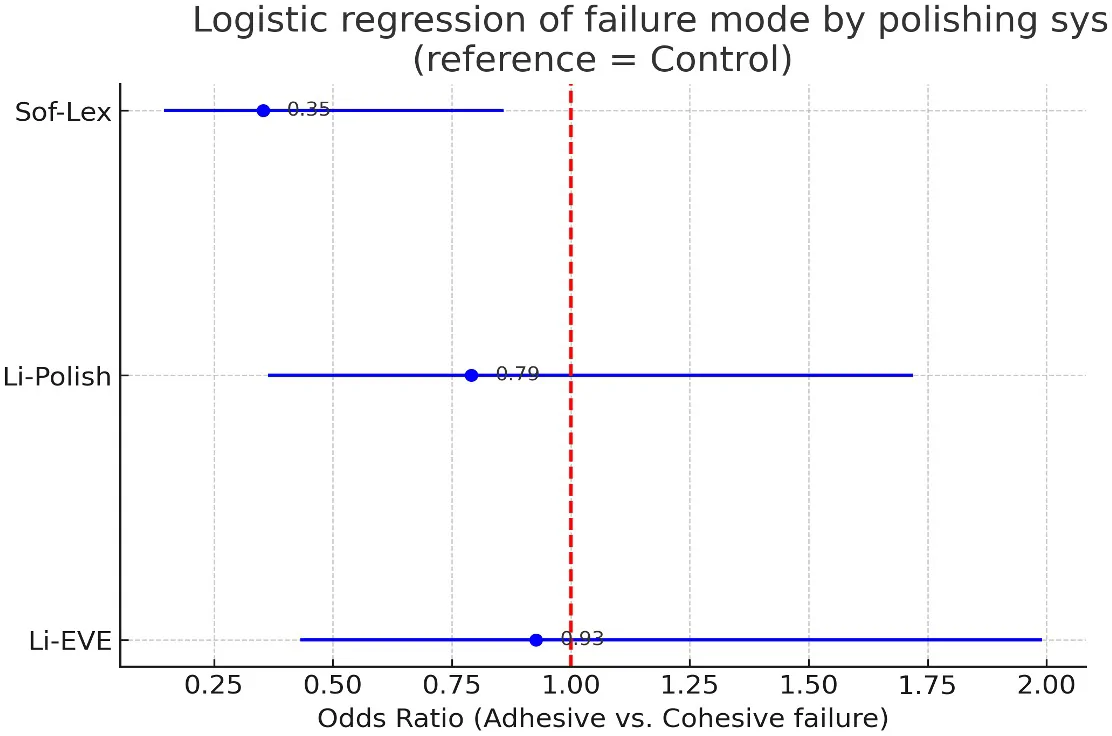
Logistic regression forest plot of adhesive *versus* cohesive failure by polishing group (reference = control). Odds ratios (OR) and 95% confidence intervals are shown; the vertical dashed line represents no effect (OR = 1). None of the polishing systems significantly altered the odds of adhesive failure.

To further confirm the findings of this study, a logistic regression model was created with the dependent variable being failure type (adhesive = 1, cohesive = 0) and polishing group as the independent variable. Using the control group as the comparator, both Li-EVE (odds ratio (OR) = 0.94, 95% confidence interval (CI) [0.42–2.08], *p* = 0.88), Li-Polish (OR = 0.75, 95% CI [0.33–1.71], *p* = 0.50), and Sof-Lex (OR = 0.39, 95% CI [0.16–0.96], *p* = 0.062 after adjustment) had no statistically significant difference from the control group. The overall model did not have a better fit than the null model (Likelihood Ratio *χ*^2^ = 6.08, *p* = 0.11). A forest plot of odds ratios with 95% confidence intervals is shown in Fig. 9, where all confidence intervals intersect the line of no effect.

**Table 2:** Pairwise *post-hoc* comparisons (Games–Howell test).

**Comparison**	**Mean difference (MPa)**	**95% CI lower**	**95% CI upper**	***p*-value**	**Interpretation**
Control–Li-EVE	−2.82	−7.51	1.87	0.23	NS
Control–Li-Polish	−7.76	−12.23	−3.28	<0.001	Significant
Control–Sof-Lex	−8.21	−12.68	−3.73	<0.001	Significant
Li-EVE–Li-Polish	−4.93	−9.41	−0.46	0.024	Significant
Li-EVE–Sof-Lex	−5.39	−9.86	−0.91	0.011	Significant
Li-Polish–Sof-Lex	−0.46	−4.42	3.50	0.82	NS

**Notes.**

NS, non-significant at α = 0.05. Results consistent with Compact Letter Display (CLD) groupings.

**Table 3:** Failure mode distribution and statistical analysis.

**Group**	**% Adhesive**	**% Cohesive**	***χ*^2^ (*df* = 3)**	***p*-value**	**Cramér’s V**
Control	33.3%	66.7%	6.31	0.098	0.162
Li-EVE	31.7%	68.3%			
Li-Polish	28.3%	71.7%			
Sof-Lex	15.0%	85.0%			

**Notes.**

Chi-square test indicated no significant difference in failure mode distribution across groups (*p* > 0.05). Cramér’s V indicates a small effect size.

## Discussion

This *in vitro* study demonstrated that enamel surfaces finished with either a multi-step aluminum-oxide disc system (Sof-Lex) or a lithium disilicate polishing kit (Li-Polish) exhibited shear bond strength (SBS) that was statistically significantly higher than both unpolished controls or the Li-EVE burs, however, the Sof-Lex and Li-Polish exhibited SBS that is statistically similar. The failure modes were predominantly cohesive across all groups. This is in line with the overall idea that the pre-bond surface state; microtopography, cleanliness, and surface free energy conditions adhesive wetting and tag formation, not just the altered adhesive chemistries (Perdigão, 2020; Papa et al., 2025). The observed differences in bond strength are consistent with established adhesion principles linking enamel microtopography and surface energy to adhesive performance; however, these variables were not directly measured, and the mechanistic interpretation remains inferential. To confirm a causal relationship between polished surface changes and bond strength, future studies will need to directly measure surface roughness and wettability.

Our finding that Sof-Lex produces higher bond strength than control and Li-EVE is consistent with profilometry/scanning electron microscopy (SEM) work demonstrating that multi-step aluminum-oxide discs restore a more uniform, groove-minimal enamel surface than single-step rubbers or, in many cases, burs—the exact substrate that promotes adhesion wetting and a consistent etching pattern (Yao et al., 2019). Shah et al. (2019) evaluated One-Gloss, Enhance/Pogo, Stainbuster, and Sof-Lex (discs/spiral wheels) and reached a straightforward conclusion that there are significant between-system differences; the Sof-Lex approaches clustered among the most effective for controlled finish (*in vitro* conditions). In the orthodontic clean-up literature there is general consensus that although no method restores enamel to pristine baseline, disc sequences repeatedly mitigate deep scratching and heterogeneity which can introduce compromising properties to bonding (systematic/clinical comparisons, and narrative syntheses) (Janiszewska-Olszowska et al., 2014; Yousry, Abolgheit & Kassem, 2024). Finally, clinical split-mouth work shows Sof-Lex discs and Sof-Lex Spiral Wheels provide indistinguishable outcomes for surface roughness and colour stability, thus, consistently reinforcing disc-based protocols as a dependable baseline (Pinzan-Vercelino et al., 2021). Mechanistically, these findings align with enamel adhesion principles indicating that durable bonding depends on surface microtopography and wettability, thereby supporting disc-based polishing protocols as a reliable baseline (Perdigão, 2020; Pinzan-Vercelino et al., 2021).

Our finding of equivalency in SBS between Sof-Lex and Li-Polish implies that some lithium-disilicate/ceramic polishing kits can simulate enamel topographies within the same “sweet spot” for etch-and-rinse universal adhesives, if used with light pressure, irrigation, and proper sequencing. Fan, Chen & Huang (2017) showed that when grit, time, and irrigation were controlled, different sequencing of finishing became equivalent in surface roughness and enamel integrity (*in vitro*) regardless of differences in working time. *In vivo*/clinical work suggests that multiple finishing protocols (*e.g.*, Sof-Lex discs *vs* Spiral Wheels) can also produce practically equivalent enamel surface roughness for bonding processes when performed well and with appropriate control factors (Pinzan-Vercelino et al., 2021). Our equivalency (Sof-Lex ≈ Li-Polish) fits the same convergence narrative.

The lower shear bond strength (SBS) for Li-EVE when comparing to Sof-Lex/Li-Polish is consistent with comparative studies that have indicated that type of abrasive, particle size, and geometry of the tool can create increased depth of furrowing, or an uneven smear, which can create variability in resin-tag formation after phosphoric-acid etching. There are several studies published that show greater areal roughness (Sa, Sq) and/or enamel loss with some burs than with sequences of discs (*e.g.*, carbide/zirconia/stone *vs* discs), all while cautioning against treating “two-step rotary” as a single entity (Ahrari et al., 2013; Thawaba et al., 2023). Reviews and clinical assessments suggest similar variability and in many cases position disc-based protocols as the safest and most predictable for recreating surface for bonding while limiting removal of enamel (Melvin et al., 2021; Sana et al., 2023). This finding likely reflects to assume our finding (Sof-Lex ≈ Li-Polish > Li-EVE ≥ control) reflects some texture-dependence effect.

Pre-bond interventions can also change the surface free energy. In a controlled study by Tamura et al. (2017) air-powder polishing showed to modulate both the SBS and the surface free energy of universal adhesives; glycine powder showed lower (less negative) change than sodium bicarbonate. While our study had no air-polish, even with no air-polish, their findings continue to reiterate the substrate-first principle in contemporary adhesion synthesis: a durable enamel bond benefits from the depth of etched microporosities and a consistent, clean, high-energy surface which provides a wetting and infiltration medium for the polymer to settle (Perdigão, 2020). This interpretation is consistent with established adhesion theory; however, as surface characteristics were not directly measured, these explanations should be considered inferential rather than definitive. Direct evaluation of surface roughness, wettability, and interfacial morphology would be required to confirm these proposed mechanisms.

Although there were differences in the between-group SBS, the failure distribution was primarily cohesive and not statistically different—the finding is also noted in conventional SBS testing in the short-term time frame when a protocol for SBS was applied, and the interfacial strength exceeded a certain threshold, fracture occurs into the composite or tooth (“ceiling effect”). More recently, studies comparing bur types (zirconia *vs* tungsten-carbide *vs* stone) and clean-up methods report similar findings: groups can be different in terms of roughness impressions, but show similar failure sites during immediate testing (Thawaba et al., 2023). Our chi-square and logistic regression analyses support this finding.

The predominance of cohesive failures suggests that bond strengths approached the cohesive limits of the substrate, indicating a potential ceiling effect inherent to shear bond strength testing. This may reduce sensitivity in detecting subtle interfacial differences between groups. Accordingly, complementary methods such as microtensile or fatigue-based testing may provide greater discriminatory capability.

For clinicians considering how to promote enamel–composite adhesion with an etch-and-rinse universal protocol, our data and the wider literature support two disciplined finishing choices: a multi-step aluminum-oxide disc option (*e.g.*, Sof-Lex) or a lithium-disilicate/ceramic polishing kit with an appropriate sequence. Both (multi-step aluminum-oxide discs and a lithium-disilicate/ceramic polishing kit) can create an enamel surface compatible with adhesion and, in our model, were superior to an unpolished control and a two-step rotary option; the advantage of the disc-based regimen with respect to smooth, minimal roughness enamel has been demonstrated previously in both profilometry/SEM and clinical split-mouth studies (*e.g.*, Sof-Lex discs *vs* Spiral Wheels), and predictably more favorable bonding outcomes (Pinzan-Vercelino et al., 2021; Thawaba et al., 2023).

In the clinical setting, do not assume that all “two-step rotary” systems can be used interchangeably; in comparative studies, differences in enamel roughness and integrity were shown across burs (zirconia, carbide, stone), prompting a word of caution with respect to the efficiency of a substitution choice leading to deeper tracks, or debrided, irregular smear (the smear inhibit wetting and micromechanical interlocking), when possible, either carryout local assessment (*e.g.*, under loupe, or engagement of a profilometer), ideally use a validated disc approach (Ghaleb et al., 2024; Yousry, Abolgheit & Kassem, 2024).

Ultimately, all adoptions should conform to mechanisms that minimize the surface free energy and detritus upon bonding: avoid rough powder-polishing or “aggressive” pre-treatments that were shown to negatively affect the adhesive bond strength of universal adhesives (glycine is less damaging than sodium bicarbonate when used for pre-treatment), and adhere to disciplined, effective finishing parameters: finish the sequence, use irrigation, and apply light pressure to enamel near the “sweet spot” for adhesive wetting; emerging evidence also indicates specific application-related details of universal adhesives (ex. passive application, etch-and-rinse method) effecting enamel bond strength to enamel, stressing the need for standardizing all aspects of practice (Tamura et al., 2017).

This investigation provides a number of methodological advantages that improve its internal validity and applicability. First, the sample size was considerable (*N* = 240 teeth; *n* = 60/group), creating the potential for sufficient power to detect between-group differences and mitigate imprecision of effect estimates. The experimental procedure was closely standardized: enamel windows were prepared in an identical fashion, finishing/polishing specifics were delineated for each system (sequence, exposure duration, rotational speed, and irrigation), and bonding was accomplished using a single universal adhesive under the same etch-and-rinse protocol, before composite application and light-curing parameters systematization. Mechanical testing proceeded under a clear standardized protocol (24-h water storage—to a 37 °C temperature, shear loading at one mm/min); failure modes were classified under magnification which permits the bond-strength data to be evaluated alongside interfacial behavior. Statistical analysis not only provided omnibus testing but also provided statistical comparisons between levels of variance and effect size for the primary outcome; failure-mode comparisons were examined with *χ*^2^ (using Cramér’s V)—then further explored with logistic regression—ultimately producing a clear, clinically useful interpretation of results. In summary, the overall sample size, stringent procedural controls, and layered statistics support the conclusion that the observed rank order among finishing systems represents true performance differences rather than chance errors.

The primary null hypothesis was rejected based on findings from the research with statistically significant differences between shear bond strength of the polishing systems. However, the secondary null hypothesis was not rejected because there were no statistically significant differences found in the failure mode distribution between any of the groups based on the experimental protocol.

The *in vitro* study should be interpreted considering the limitations of their use in replicating the complexities of the oral environment. Many factors including pellicle formation, biofilm activity, pH, thermal cycling and cycle mechanical loading that could affect long-term bond strength were not able to be simulated. More importantly, due to the lack of thermocyling and fatigue loading, the results cannot provide an accurate assessment of hydrolytic degradation, interfacial fatigue or marginal deterioration over time.

Furthermore, only one universal adhesive system and bonding regimen was utilized, therefore the generalization of the data does not readily extend to all possible adhesive regimens and clinical situations. In addition, due to the lack of direct surface characterization, quantitative analysis of surface roughness and free surface energy were not performed; therefore, the empirical relationship between polishing protocols and adhesive performance is inferred. Given that the sensitivity of enamel bonding is based upon the microtopography and wettability of the substrate, the inability to perform a profilometric analysis, contact angle measurements or high-resolution images limits the determination of structure–property relationships.

Additionally, enamel surfaces were mechanically reduced before polishing and bonding for the purpose of creating a standardized bonding substrate and to minimize variability associated with natural enamel morphology. This method of mechanical reduction improves the internal validity of the study, but does not accurately represent the typical bonding procedure used to bond intact, unprepared enamel which occurs frequently during clinical procedures. Removing the outer aprismatic layer of enamel may alter surface characteristics and etching characteristics of the enamel that can potentially impact adhesive performance. As a result, the study’s findings should be interpreted as reflecting the bonding of prepared enamel substrates, therefore caution is suggested in extrapolating these study results to clinical procedures involving unprepared enamel. In addition, only premolar teeth were included in this study. Variations in enamel thickness and prism orientation across different tooth types may influence bonding behavior and shear bond strength outcomes. Therefore, the findings may not be directly generalizable to other teeth, such as molars or anterior teeth, where enamel morphology differs. Future studies should consider evaluating multiple tooth types to better understand the influence of enamel structure on adhesive performance. Bond strength data were not stratified according to failure mode, and failure analysis was performed descriptively; therefore, potential differences in bond strength associated with specific failure patterns were not evaluated.

Future studies should include the use of artificial aging procedures along with advanced surface metrology and utilize multiple bonding systems to obtain a better understanding of the relationship between the polishing process and adhesion performance in a clinically relevant manner.

## Conclusions

In this *in vitro* study, disciplined finishing methods conditioned enamel for improved adhesion to the composite restoration, multi-step aluminum-oxide discs (Sof-Lex) and a lithium-disilicate polishing protocol (Li-Polish) resulted in significantly greater shear bond strength than any no polishing or Li-EVE, with Sof-Lex ≈ Li-Polish. All failure modes noted in this study were predominantly cohesive and no group was significantly different, inferring that all groups reached similar limits of interfacial strength. Recognizing that clinical considerations differ from *in vitro* methods, and accepted clinical protocols need to be better established, using multi-step disc or validated ceramic-polishing protocol, that includes irrigation and using light pressure and full sequences as any adhesion-based choice is defensible to use. However, within the short duration of the experiment, the consideration of potential long-term viability of enamel surfaces formed by polishing will need to be considered in empirical follow-up studies of durability, which are warranted based on the present study’s results.

## Supplemental Information

10.7717/peerj.21549/supp-1 Supplemental Information 1 Raw Data

10.7717/peerj.21549/supp-2 Supplemental Information 2 STROBE Checklist

## Additional Information and Declarations

### Data Availability

The following information was supplied regarding data availability: The raw data is available in the Supplementary Files.
